# Integrating the spiritual dimension in long-term care: a mixed methods evaluation of a multicomponent intervention for nursing home teams

**DOI:** 10.1186/s12912-025-04089-3

**Published:** 2025-12-12

**Authors:** Niecky Fruneaux-van Amerongen, Anke Persoon, Ewald Bronkhorst, Yvonne Engels

**Affiliations:** 1 Department of Spiritual Care, Liemerije, Hunneveldweg 12, Zevenaar, 6903ZN The Netherlands; 2 Department of Primary and Community Care, Radboud University Nijmegen Medical Center, Nijmegen, The Netherlands; 3 Department for Health Evidence, Radboud University Nijmegen Medical Center, Nijmegen, The Netherlands; 4 Department of Anaesthesiology, Pain, and Palliative Medicine, Radboud University Nijmegen Medical Center, Nijmegen, The Netherlands

**Keywords:** Person-centered care, Meaning in life, Spiritual care, Competencies, Nursing home care, Spirituality

## Abstract

**Background:**

Moving to a nursing home has a major existential impact on the lives of residents and their relatives. Healthcare professionals who have daily contact with residents have many opportunities to pay attention to existential and spiritual issues. To support them, the Dutch multidisciplinary guideline on existential and spiritual aspects of palliative care was developed. However, many healthcare professionals report a perceived lack of competence in this domain and it remains unclear what they need to effectively translate the theory and tools from the guideline into daily practice. The multicomponent intervention ‘Insight into meaning’ (Zicht op zingeving) was developed, to facilitate the integration of the spiritual dimension into the daily practice of healthcare professionals. It consisted of structured training sessions, individualized on-the-job coaching and team intervision meetings. This study aimed to examine the effects of the intervention by exploring how healthcare professionals assess their spiritual competencies before and after the intervention, the focus of their coaching questions and learning outcomes during individual on-the-job coaching, and how they apply spiritual skills in daily practice.

**Methods:**

A mixed method experimental design was used to examine the effects of a multicomponent intervention on two wards of a Dutch nursing home. The study included 44 healthcare professionals: primarily certified nurse assistants and client support workers. Participants were recruited via non-probability volunteer sampling and completed the Spiritual Care Competency Scale (SCCS) before and after the intervention. Quantitative data were analyzed using paired samples t-tests. Qualitative data were collected via coach registration forms and analyzed using thematic and deductive content analysis to explore participants’ coaching questions, learning outcomes, and observed spiritual competencies.

**Results:**

SCCS total and subscale scores showed statically significant improvements for pre- to post intervention (n=19; Δ0.50). Participants foremost frequently sought coaching to strengthen their role in addressing the spiritual dimension in interactions with residents. An increased awareness of their personal role in such encounters was commonly reported. Moreover, participants who did not articulate specific coaching questions appreciated the individual feedback. The observations provided valuable insight into the practical application of spiritual competency: particularly in the areas of aligning, connecting and deepening. A variety of responsive actions were noted, when healthcare professionals pay attention to what matters most to residents

**Conclusions:**

The multicomponent intervention led to improvements in self-reported spiritual competencies among healthcare professionals. Both the training sessions and individualized on-the-job coaching contributed to heightened awareness of the spiritual dimension in daily practice. The study provided valuable insight into how theoretical frameworks and practical tools related to the spiritual dimension can be effectively integrated into the daily practice of healthcare professionals working in a nursing home setting.

## Background

Moving to a nursing home has a major existential impact on the lives of residents and their relatives [1]. People who move to a nursing home leave their familiar surroundings behind, resulting in significant changes in personal relationships and the wider social network. Within the institutional setting of a nursing home, residents are confronted with constantly changing healthcare professionals and fellow residents whom they did not chose. Additionally, residents contend with one or more chronic conditions that compromise their mobility and autonomy, reinforcing the realization that the nursing home will likely be their final place of residence. Especially the most frail older adults enter a nursing home, often during the final stage of life [2, 3]. Consequently, they may come to terms with increasing dependency and imminence of death. Even excellent care cannot take away this existential upheaval. Addressing these challenges requires a high level of sensitivity from healthcare professionals to attune to it, and an ability to attune to the residents’ deeper needs. Crucially, care on the spiritual dimension in this context is less about problem solving (doing) and more about presence, attentiveness and relational depth (being) [1].

Numerous existential questions and themes emerge in the context of nursing home care, requiring attentive and empathic listening and engagement. While spiritual caregivers can provide support and guidance during specific encounters, healthcare professionals have daily contact with residents, and can recognize and respond to spiritual concerns as they arise in everyday interactions. However, because individuals do not typically articulate their deepest concerns, these issues can be difficult for healthcare professionals to identify [4]. Many healthcare professionals report a perceived lack of competence in this domain [4]. Nevertheless, it is important to explore the areas in which healthcare professionals may already be demonstrating unconscious competence in addressing the dimension of spirituality, as their daily work consistently exposes them to the existential impact that living in a nursing home has on residents and their relatives. The basic educational programs for healthcare professionals pay very little or no attention to the spiritual dimension of care, or to how to recognize and respond to issues and questions that may concern someone.

For these reasons, the ‘Dutch multidisciplinary guideline Existential and Spiritual Aspects of Palliative Care was developed to provide support for healthcare professionals in integrating the spiritual dimension into daily healthcare [5]. With the help of practical tools, like the Mount Vernon Cancer Network questionnaire [6], the guideline offers guidance for healthcare professionals in identifying and exploring spiritual issues with their palliative patients during daily practice.

An important first step in addressing spiritual needs is recognizing and exploring the cues that residents and their relatives give, which might reflect what matters most to them [4]. Up to now it has been unclear what healthcare professionals require to effectively translate the theory and tools from this guideline into their daily practice. For that reason, we developed a multicomponent intervention to increase awareness of the spiritual dimension. The aim of this study was to examine the effects of the intervention. Specifically, we examined how healthcare professionals assess their spiritual competencies before and after the intervention, the focus of their coaching questions, and the learning outcomes during individual on-the-job coaching, and how they apply spiritual skills in daily practice. 

## Methods

### Design

**Fig. 1:**
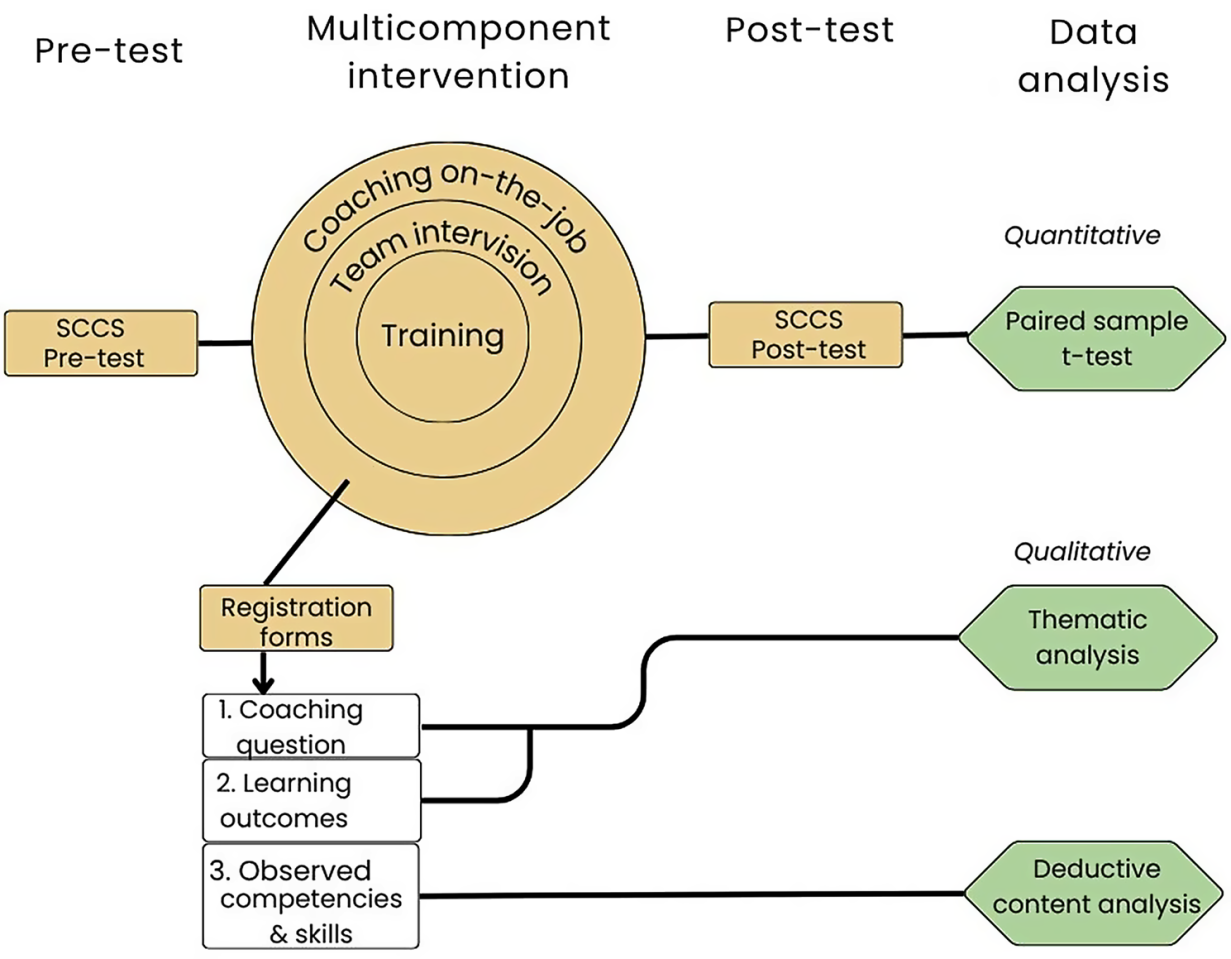
Overview mixed method research design

A mixed method experimental study was performed using quantitative and qualitative data to examine the effects of a multicomponent intervention. Figure 1 shows an overview of the research design.

### Intervention

The intervention ‘Insight into meaning’ (Zicht op zingeving) was developed to facilitate the integration of the spiritual dimension into the daily practice of healthcare professionals. The spiritual dimension was defined as “*a dynamic dimension of human life relating to the way individuals (individually and in community) experience, express, and seek meaning, purpose, and transcendence, and the way they connect to the moment, to self, to others, to nature, and to the significant and/or sacred*” [7]. This definition encompasses an individual’s sense of meaning and purpose in life, which may vary from person to person and evolve over the course of a lifetime. The *Insight into meaning* intervention was developed from a previous action research study, shaping the operationalisation of the spiritual dimension, the selection of the theoretical concepts and components incorporated into the intervention. In this previous action research study, professionals from a wide range of roles within the nursing home participated, from nurse assistants to members of the board.

The attention that healthcare professionals devote to the spiritual dimension in daily practice was operationalized as the ability to see, hear and adequately respond to what matters most to someone. The theoretical foundation for the training was based on four concepts, which are also outlined in the Dutch multidisciplinary guideline Existential and Spiritual Aspects of Palliative Care: inner space, listening, being present with attention, and layers of meaning (see Box 1). The intervention consisted of three components: a training [8], individual on-the-job coaching and team intervision meetings (see Box 2). The intervention was carried out by five coaches who work in the nursing home as a spiritual caregiver, vocational trained nurse and vocational nurse assistant. The coaches received bi-monthly supervision from two experienced trainers with a background in spiritual care.

**Box 1:** Theoretical basis of the training

*A: Inner space*
The concept of inner space is frequently used in palliative care to describe the state of mind of (palliative) patients [9]. Inner space refers to one’s attitude towards existential and spiritual issues in life. Having a lot of inner space is ‘a state of mind that enables one to be aware of one’s actual thoughts and feelings without being overthrown or swept away by them’ [9]. While originally applied to patients, this concept is also helpful and important for healthcare professionals. Cultivating inner space requires mindfulness to allow professionals to become aware of their own existential and spiritual issues as well as those of their patients or residents, without getting emotionally overwhelmed. When sufficient inner space is present, healthcare professionals’ capacity to remain accepting, open and empathic increases, enabling them to respond with appropriate emotional attunement. Fostering awareness of one’s own inner space was a key element of the intervention.
*B: Being present with attention*
Being present with attention is essential for establishing meaningful contact with residents [10, 11]. This approach is grounded in the principle that the patient is the expert on their own life and experiences, including how they perceive their situation. It differs from an action-oriented attitude, in which healthcare professionals primarily focus on interventions based on their own expertise. Both approaches are valuable within the care relationship. During the coaching session the way participants’ ability to be present with attention was observed.
C: *Listening*
The training placed strong emphasis on the development of listening skills as attentive listening forms the foundation of all engagement with the spiritual dimension of care. Spiritual themes do not usually appear through formal screening or checklists but arise naturally in conversation when there is trust and connection. This requires ‘listening with space’: for the other person and for oneself as a dialogue partner. In what concerns people, themes automatically emerge, which can be explored with them. Consequently, the concepts of being present with attention and inner space are the foundation of these listening skills [8].
D: *Layers of meaning*
A single statement from a client can have different meanings [5, 12]. Weiher has worked this out in a model and states that you can connect to different layers in contacts:
1. Factual: Physical, embodied meaning. 2. Emotional: psychological, felt meaning 3. Biographical: social, socio-cultural meaning 4. Existential: spiritual, existential meaning.

**Box 2:** Components of the intervention Insight into meaning components of the intervention

1) Training
Aimed to familiarize participants with a different listening attitude, conversational skills and tools. It was based on practical experiences of two senior professionals within the spiritual dimension and was closely related to the practical tools and insights of the Dutch guideline. Participants were asked to rate their overall satisfaction with the training on a scale 0–10.
2) individual coaching on the job
This took part in the months after the training had taken place. Participants worked on individual questions and skills in the context of their own profession. All participants were asked whether they had a specific question they wanted to focus on during the coaching sessions. The coach would accompany the participant for about 1,5–2 hours during a regular workday and gave feedback during and after the session.
3) Team intervision meetings*
The participants could discuss the questions they had about a work situation in relation to the content of the training, collectively learning from these situations. Each participant enrolled in at least two intervision meetings on their own ward, also during the months after the training had taken place.

*Data from the intervision meetings are not reported in this article

### Setting and inclusion of participants

The study was conducted on two wards within a medium-sized nursing home in the eastern part of the Netherlands which has a total capacity of 469 beds. One ward housed primarily residents with somatic diseases (18 residents) and one ward with residents with psychogeriatric diseases (36 residents). The institution employs a diverse range of healthcare professionals ranging from client support workers to nurses, with the majority (44–56%) being certified nurse assistants. Additional professionals, working in the department include doctors, physiotherapists, occupational therapists, speech therapists, psychologists, social workers and spiritual caregivers. Most healthcare professionals in the nursing home are women (91%^1^).

Participants were recruited using a non-probability volunteer sampling method. Participants received financial compensation for the time invested outside regular working hours. Inclusion criteria were being employment at one of the participating wards and providing informed consent. Although the initial aim was to engage approximately 2/3 of the healthcare professionals, only 1/3 of them registered to participate (*n* = 44). We did not use a control group in this study.

### Data collection

Data collection took place between October 2022 and March 2024. Information and informed consent forms were provided and obtained beforehand. Two sources of data were administered: the Spiritual Care Competence Scale (SCCS) for self-evaluating spiritual competencies and a registration form filled out by the five coaches.

### Quantitative data collection

Before and after the intervention the participants completed the SCCS. The SCCS consist of 27 questions rated on a 5-point Likert scales ranging from 1 (totally disagree) to 5 (totally agree), including the following subscales: assessment and implementation of spiritual care; professionalization and improvement of the quality of spiritual care; personal support and patient counselling; referral to professionals; and attitudes towards patients’ spirituality and communication [13]. As the SCCS was originally developed for the hospital context, several questions were adapted to better reflect the nursing home setting. Four questions were excluded due to overlap (s13, continue daily spiritual customs and rituals) or lack of relevance for healthcare professionals working in nursing homes. (s5 coordinating care, s11 contributing to quality of spiritual care and s12 addressing work related problems) Items from the original Communication scale and the Attitude towards Patient’s Spirituality scale were combined into a single composite measure: ‘Attitude towards patient’s spirituality and communication’.

### Qualitative data collection

A registration form was completed by the five coaches to document observed competencies. This registration form was based on the theoretical basis of the training. The coaches were briefed beforehand and were familiar with the aspects of the registration form as they played an important role in the training (Box 1).

After each individual on-the-job coaching session, the coaches used this registration form. They documented the participant’s coaching questions, as stated at the beginning of the session, what was learned from the sessions as well as the observed competencies and skills (having inner space, listening, being present with attention, using the layers of meaning, see Box 1).

### Data analysis

Quantitative data were analyzed using paired samples t-tests. Mean satisfaction scores and standard deviations were calculated for the pre- and post-tests. Paired samples t-tests were used to compare sum scores of the scales of the pre- and post-tests of the SCCS before and after training. All quantitative analyses were performed using SPSS version 29.

Qualitative data were coded line-by-line via Atlas.ti version 9. Thematic analysis [14] was applied to explore the questions participants stated at the beginning of the on-the-job coaching sessions [14].

Observations of spiritual skills during individual on-the-job coaching sessions were analysed via deductive content analysis [15]. Data were categorized by NF and AP based on a prior study in which skills related to the spiritual dimension were identified through reflexive thematic analysis of interviews with residents and residents’ relatives [16]. This framework included three themes with eight categories: aligning (being present with attention and recognizing individuality), connecting (attuning approach, attuning communication and building a care relationship) and deepening (deepening contacts and recognizing life-questions). 1) *Aligning* revolves around the skills of tuning in as a process and an attitude of inquiry: who is this person? What does he/she want or find important? A key element of alignment is the inner space of healthcare professionals. 2) *Connecting* involves fostering mutual involvement between healthcare professionals and residents and their loved ones. Something happens between two people in the (care)relationship. 3) *Deepening* encompasses the ability to explore underlying meaning of an expression when recognizing cues related to the spiritual dimension. Deepening entails that healthcare professionals dare to open up, are curious about the spiritual dimension, different layers of meaning, can ask inviting questions and explicitly name their observations in their contact with residents. Categorizations were discussed among NF, AP and YE until consensus was reached. In cases where observations did not conform to the current structure, new themes and categories could be introduced.

### Ethical considerations

This study was conducted in accordance with Dutch law and Good Clinical Practice guidelines and the Declaration of Helsinki. As the study involved the observation of routine care without subjecting participants to treatment or requiring specific behaviours, the Medical Research Ethics Committee Oost-Nederland determined that this study was not subject to the Medical Research Involving Human Subject Act (2022–13622). Written informed consent was obtained from all participants. To ensure the anonymity of both participants and clients, identifying client characteristics, including quotes, were modified to prevent recognition.

## Results

This section reports on the outcomes of the intervention. The results are presented in four parts. The first part presents a description of the demographic and professional characteristics of the participating healthcare professionals (Table 1), followed by an overview of participant inclusion across the various components of the intervention and the corresponding analyses (Table 2). The second part presents the outcomes of the self-evaluation of spiritual competencies, as measured by the Spiritual Care Competence Scale (SCCS). Pre- and post-intervention scores are compared to assess changes in perceived competencies (Table 3).

**Table 1:** Characteristics of participating healthcare professionals

Variable		N (%)
Total		44 (100)
Profession	Vocational trained Nurse	3 (6.8)
	Certified nurse assistants	21(47.7)
	Client Support Workers	10 (22.7)
	Social worker	2 (4.5)
	Psychologist	2 (4.5)
	Physician assistant	1 (2.3)
	Physical therapist	1 (2.3)
	Occupational therapist	1(2.3)
	Team coordinator	1(2.3)
	Activity supervisor	1(2.3)
	Spiritual caregiver	1(2.3)
Ward	Mainly somatic conditions (18 beds)	17(38.6)
	Mainly psychogeriatric conditions (36 beds)	27 (61.3)
Gender	Female	42 (95.5)
	Male	2 (4.5)

**Table 2:** Inclusion of participants in different components of the intervention

Components of the intervention	Participants
3) Training First group: online sessions: 4 sessions of 1.5 hour. Second group: live sessions: 3 of 3 hours. Overall satisfaction score: 8.5	Total: *n* = 44 First group: *n* = 20 Second group: *n* = 24 Evaluation: *n* = 44
3) Individual coaching on the job 2 sessions of 1.5–2 hours First group: 3 coaches Second group: 5 coaches	Total: *n* = 44 Analysis: *n* = 13
3) Team intervision meetings*: 2 live sessions of 1 hour	First group: 13 meetings Second group: 9 meetings Total: 22 meetings took place

*Qualitative data from the intervision group are not reported in this article

**Table 3:** SCCS sum-scores on pre-test and post-test

SCCS and subscales (1 totally disagree to 5 totally agree)	Pre-test mean (*n* = 44)	SD	Post-test mean (*n* = 19 *)	SD	Δ (pre-post -test) (*n* = 19)	95%-CI	*P* = value
Assessment and implementation of spiritual care (4 items)	3.5	0.6	3.92	0.6	0.66	0.40–0.92	< 0.001
Professionalization and im- proving the quality of spiritual care (4 items)	3.1	0.76	4.45	0.83	0.72	0.38–1.07	< 0.001
Personal support and patient counseling (6 items)	3.43	0.51	3.9	0.56	0.53	0.27–0.78	< 0.001
Referral to professionals (3 items)	3.46	0.67	3.82	0.55	0.40	0.12–0.68	0.007
Attitude towards patient’s spirituality and communication (6 items)	4.43	0.41	4.62	0.37	0.26	0.02–0.51	0.035
**Total (23 items)**	**3.65**	**0.40**	**4.18**	**0.46**	**0.50**	**0.31–0.69**	** < 0.001**

* due to a technical error only participants of the second cycle could be paired

The third part focuses on the coaching questions and learning outcomes during on-the-job coaching. Thematic analysis of the coaching questions is presented, followed by qualitative findings what was learned from the individual coaching session. Illustrative quotes are provided to exemplify the skills participants worked on during the coaching process (Table 4). The final part reports on the observed spiritual care skills during on-the-job coaching. These observations are categorized according to three overarching themes—aligning, connecting, and deepening—based on a previously established framework, with additional subcategories identified inductively from the data.

**Table 4:** Examples of quotes about the skills participants worked on

a) How a participant became more aware of her own role in contacts:
*‘When a colleague asked if the participant could stay longer, the participant took care of their own inner space by asking for some time to think about this decision. When we talked about how to take care of your own inner space, the participant recognized having made a more conscious choice that positively affected their inner space, even amidst the hustle and bustle.’* (Coach C, participant 22)
b) Participants’ awareness of life questions and how these questions are also part of their personal daily life:
*‘The participant gained more awareness that there are almost always ‘hidden’ questions of meaning during a conversation. Not only with a resident but also with themselves (For example, calling with one’s own child before bedtime during a shift, as this participant does)* [as they discussed the hidden life-question this call had for the participant].’ (Coach B, participant 8)
c) Participants’ awareness of their own communication:
*‘The participant was not aware that they were already naming emotions* [which had been their coaching question]*. The participant was happy to hear it.’* (Coach C, participant 14)
d) appreciation of individual feedback:
*‘It provides recognition but also space to reflect on how you do your work. I think it is nice to hear positive points and receive constructive comments.* (Coach C, participant 11)

### Participation and inclusion of healthcare professionals

This section outlines who participated in the intervention and how they were involved, offering context for interpreting the results. Table 1 summarizes the demographic and professional profiles of the participating healthcare professionals. Table 2 presents inclusion of participants across the intervention’s components and indicates which data sources were used in the subsequent analyses.

### Self-evaluation of spiritual competencies

This section presents the results of the self-assessment conducted using the Spiritual Care Competence Scale (SCCS). Changes in perceived competencies before and after the intervention are examined to assess its impact on participants’ spiritual care competencies.

The response rate was 100% for the pretest (*n* = 44) and 43% for the post-test (*n* = 19). Table 3 shows the outcomes of the pre- and post-tests for self-evaluations with the SCCS. The sum scores of the scales and subscales all significantly increased, indicating that the intervention improved spiritual competencies in these aspects.

### Individual coaching questions and learning outcomes

This section presents findings from the coaching questions during individual on-the-job coaching. A thematic analysis of participants’ coaching questions is followed by qualitative insights into the learning outcomes.

Most of the coaching questions were about the participant’s own role in the conversation with the resident: giving more space (25%), deepening contacts (25%) and being present in the moment rather than focusing on problem solving (25%). Coaching questions concerning the layers of meaning (17%) always involved increasing awareness of these layers in daily practice. Finally, the theme of balancing attention between the group and the individual (8%) reflected an internal struggle among healthcare professionals to practice keeping more space in contacts, while several simultaneously being aware of the needs of other residents.

Following the coaching session, the coaches reflected on what the participants had learned from the training and what might help them to further develop their skills. The observations indicated that individual coaching was valuable in increasing healthcare professionals to gain more awareness of a) their own role, b) questions about meaning in life and c) their own communication style. Additionally, they often expressed d) their appreciation for receiving individual feedback to support their development, regardless of whether they had formulated a specific coaching question or not. Table 4 shows examples of quotes about the skills participants worked on.

### Observed spiritual skills

This section presents findings from on-the-job coaching observations. Spiritual care practices were categorized into three overarching themes—*aligning*, *connecting*, and *deepening*—based on an existing framework, with additional subthemes identified inductively from the data.

Of the 44 participants, 13 participants were included in the analysis based on the quality and completeness of the registration forms, as well as representation across the five coaches and the two wards. The observations of spiritual skills during individual on-the-job coaching aligned well with the three preformulated themes, derived from a previous study. (Fruneaux - van Amerongen et al., 2024). Within each theme, one or two categories were added, resulting in the following: 1) aligning; (managing inner space, being present with attention, recognizing individuality and listening), connecting (attuning approach, attuning communication and building a care relationship) and deepening (deepening contact, recognizing life-questions and layers of meaning.

#### Aligning

Within the theme alignment, four categories of skills were observed: a) managing inner space, b) being present with attention, c) recognizing individuality and d) listening, of which managing inner space and listening were new categories added to the original theme Aligning.

*A) Managing inner space* influences the ability to align and attune to the person with whom one is in contact. In three forms, the participants reported to have a certain effusion of calmness and composure. Additionally, twelve examples of ways to expand inner space were reported that could be captured in three underlying actions: sharing experiences, taking a moment for oneself and realizing that one is not alone. Conversely, diminished inner space was reported in three forms: personal challenges in life, control tasks under time pressure and increased absenteeism.

A number of things that happened gave the participant recognition about their own situation. The participant did not take over the conversation with a personal story, but did share what this is like for them. In a careful, calm way. In doing so, the participant revealed parts of themselves and managed their inner space. This helped to connect with the resident’ (Coach B, participant 8)

*B) Being presen*t with attention was frequently reported. The observations provided more insight into both the struggles healthcare professionals face and the strategies they use to remain present with attention during moments of contact. Difficulties were particularly evident in situations where multiple urgent tasks competed for their attention, potentially drawing focus away from the resident. We observed remaining present with attention as follows: staying close, applying important information about the resident and remaining resident-oriented.

The participant sits in such a way that the residents can see they receive full attention. The participant allows silence so that the residents have time to process. The participant also provides an opening for another topic that is currently relevant in their lives. Residents seem to feel at ease with this participant (they show their emotions and share memories). (Coach A, participant 4)

*C) Acknowledging individuality* was identified in the data across five categories: alignment with individual needs; recognizing deviations from usual behaviour; alignment with possibilities and limitations; space for personal expression; and demonstrating individuality as a healthcare professional. A novel insight emerged in the fifth category, revealing how healthcare professionals utilize their individuality to foster person-to-person connections. For example, alignment with individual needs was illustrated by a situation in which a resident needed to use the bathroom:

The resident is restless and keeps pacing around the living room. The resident wants to say something but cannot express themself. A colleague seats them at the table, stating: “You must eat first”. The resident repeatedly gets up and approaches the participant, who knows the resident well, as evident in their interaction. Immediately, the participant recognizes the residents’ need to use the toilet. The resident visibly relaxes upon realizing they are understood. (Coach A, participant 4)

*D) Listening*. To explore how healthcare professionals listen to residents, listening behaviours were analysed, leading to the addition of a separate category within the theme of *Connection*. Observations revealed several strategies employed by healthcare professionals in their daily work: maintaining an attentive posture, having the same eye-level, allowing silence, utilizing nonverbal communication, showing genuine interest, sitting with the resident and letting the resident lead conversations. For example,

When the resident speaks, the participant remains silent, maintaining an attentive listening posture and allowing silences to occur, so that the resident can lead the conversation. The participant occasionally hums to signal acknowledgment and understanding, and briefly summarizes key points throughout the conversation. (Coach A, participant 1)

#### Connecting

Within the theme of Connecting, three key categories emerged: a) attuning communication; b) attuning approach; and c) building a care relationship. Connection occurs when the approach and (non)verbal communication is attuned to the resident and/or relative.

A) Attuning communication. Attuned communication involves both verbal and nonverbal exchanges. Observed behaviours include confirming comprehension; articulating clearly; engaging in nonverbal communication; summarizing; returning to topics; setting boundaries; selectively withholding responses; and explicitly stating actions and intentions.

The participant congratulates the resident on their achievements in a game but quickly notices that the stimulation is overwhelming. Still, the participant remains present, ready to respond to cues. “I see you have a dry mouth; would you like some juice?” The resident answers “Yes, please” while simultaneously gesturing dismissively’. (Coach C, participant 5)

B) Attuning approach. Healthcare professionals demonstrated adaptability by taking adequate time, incorporating physical contact and modifying their pace to suit the situation.

While dispensing medication, the participant always took time to pay attention to individual residents. Sometimes sharing a joke, offering an affectionate gesture or giving a compliment. (Coach C, participant 14)

C) Building a care relationship. Healthcare professionals fostered trust and reciprocity in daily practice. For example:

Did you see that? I was done, and I wanted to walk away, but then the resident said something else. Did you hear what they said? “I wish you all the health and happiness in your life.” The resident said it entirely of their own accord. That is truly the greatest compliment I could receive from this resident. (Coach C, participant 5)

#### Deepening

Three categories were identified within this theme: a) deepening contact; b) acknowledging life-questions; and c) exploring layers of meaning. The latter category was introduces based on observations.

A) Deepening contact in daily practice involves asking follow-up questions, providing space for personal narratives, exploring meanings and explicitly naming emotions. Newly observed behaviours, such as exploring meanings and naming emotions contributed to a more comprehensive understanding of effective deepening strategies.

What is that like for you, having been independent with a busy career, and now relying entirely on others?’ “Yes, I find it incredibly difficult. I can no longer do anything myself, you need someone else for everything” (…) “Powerless … is that how you feel?” “Yes, powerless … But you have no choice. There is nothing left to want.” (Coach C, participant 8)

B) Recognizing life-questions. Healthcare professionals rarely identified life questions. This is a specific skill that contributes to deepening. This example shows that raising awareness of life-questions can significantly impact individual colleagues and team dynamics:

I am much more aware of it now. As a result, I focus far less on solutions. Additionally, saying things aloud makes a difference. Take that resident - who suddenly became critically ill, completely jaundiced. We decided against sending them to the hospital for treatment. However, this decision was difficult for the team. Now I said aloud to colleagues: can this person be allowed to die here? Later, a colleague approached me and said, “I had not thought about it that way before.” (Coach C, participant 24)

C) Exploring layers of meaning. Observations revealed that healthcare professionals often explore layers of meaning to deepen contacts, though the spiritual layer was least reported.

The participant initiates the conversation with the “difficult resident” by commenting on the air conditioning - it was a hot day. Quickly, it becomes clear that something is bothering the resident. The participant asked, “What exactly is going on?” As the resident responded, first factual questions were asked, but during the conversation, also the residents’ emotions were mentioned: “Why are you so angry?” and “it is okay to be sad about that.” The participant gently probes further: “What makes you so sad?” (Coach C, participant 14)

## Discussion

This study aimed to examine the effects of the intervention by exploring how healthcare professionals assess their spiritual competencies before and after the intervention, the focus of their coaching questions, and the learning outcomes during individual on-the-job coaching, and how they apply spiritual skills in daily practice.

Post-intervention the self-reported spiritual competence of the participants had significantly increased. The marked improvement in the domain of “professionalization and improving the quality of spiritual care” suggests that the intervention has been particularly effective in helping participants recognize and value spirituality within healthcare. While the limited number of completed pre-post SCCS questionnaires constrains the strength of our quantitative conclusions, we interpreted these results with caution and situated them within the broader mixed-methods framework. The qualitative data in particular offer meaningful insights into the intervention’s impact and the mechanisms through which spiritual competencies were developed.

Throughout the intervention, frequent references to the spiritual dimension were noted. The questions during individual on-the-job coaching largely focused on the healthcare professionals’ role in conversations. Observations revealed numerous instances of the three spiritual skills: alignment, connecting and deepening. Although the number of analysed forms was limited to thirteen, this purposive selection – covering all five coaches and both wards – enabled thematic saturation and yielded a rich and interpretable dataset. This approach reflects a deliberate balance between analytical depth and practical feasibility and enhances transparency by capturing the diversity of coaching experiences within the study context. Additionally, various actions demonstrating attention to the spiritual dimension in daily practice were documented.

Our findings indicate that healthcare professionals address the spiritual dimension not as a separate element but as an integral aspect of their daily work, achieved through openness and awareness.

The data revealed how professionals recognize and respond to what matters most to residents and their relatives. Their ability to support the daily meaning in life of nursing home residents appeared fundamental. This insight sheds a different light on the notion of a difference between daily meaning in life and existential meaning in life.

Daily meaning in life is about the meaning of personal life, whereas existential meaning in life concerns the question of the meaning in life in general [17]. Psychological theories often differentiate between these constructs [18] with religion, the philosophy of life and spirituality serving as sources of existential meaning. However, in today’s Dutch secularized society, meaning is increasingly derived from more tangible aspects of life such as work, relationships and personal development [18].

The study identified numerous empathic and sensitive healthcare professionals demonstrating unconscious competence in addressing daily meaning in life. It deepened our appreciation for the complex, often undervalued contributions of vocational trained nurses, certified nurse assistants and client support workers in nursing homes. These findings may lead to a re-evaluation of the significance of ‘daily meaning in life’ and its connection to the spiritual dimension in healthcare. Daily meaning in life may reflect a more contextual and relational understanding of spirituality in secular care settings. Rather than being rooted in religious or philosophical frameworks, meaning-making in this context emerges from everyday interactions, shared experiences, and the affirmation of residents’ identities in the here and now. Such an interpretation resonates with contemporary perspectives that view spirituality as embedded in social relationships and daily practices, yet it also challenges more traditional theoretical models that prioritize transcendence or ultimate life purpose. By situating spirituality within the lived realities of residents and the relational competencies of care staff, this study contributes to an expanded conceptualization of spiritual care that is both context-sensitive and practically grounded. In this light, it may be argued that daily meaning in life is naturally embedded in routine care practices of healthcare professionals, whereas existential meaning may require the attention of dedicated healthcare professionals such as spiritual caregivers.

Contrary to expectations, coaches primarily provided reflection and general communication feedback rather than reinforcing knowledge and skills from the training. They had to and did attune their approach to the participants’ needs and what was happening during the observation. It resulted in numerous examples of practical skill application that enhanced alignment, connection and deepening communication. The observation that coaches primarily facilitated general reflection and communication rather than reinforcing specific training content, suggests that guided reflection may be a key mechanism in developing spiritual competence, complementary to the training intervention. This may indicate that reinforcing spiritual competence relies less on the direct transmission of content and more on the ability to engage in meaningful, context-sensitive reflection. In this light, the coaches’ role as a facilitator of dialogue and introspection becomes central to the learning process, highlighting the importance of creating space for personal meaning-making and experiential learning in professional development. This aligns with research on effective workplace learning, closely tailoring learning to the situation and learning preferences of individual healthcare professionals [19–22]. Based on these insights, we recommend workplace learning initiatives.

Many participants noted the personal and professional impact of the intervention. It is crucial to reflect on the implications of expecting spiritual competency from healthcare professionals. We noticed that healthcare professionals became more open and receptive to their own existential issues, pain, sadness and disappointment in life and that of others. Although it is known that healthcare professionals experience greater job satisfaction when they see that they can mean something to someone, this is commonly used as an argument for integrating spiritual care in daily practice [23, 24], we simultaneously observed that it demands a lot of healthcare professionals to be confronted with existential pain. We need to pay attention to the impact on individual healthcare professionals. This finding was reported by other studies with on-the-job coaching on the spiritual dimension [25, 26]. A similar conclusion was reported in a German study that entailed a 40-hour training program for healthcare professionals in nursing homes, also highlighting the importance of developing team-spirit [27–29]. We advocate for a tailored approach that considers healthcare professionals’ individual needs, abilities and motivation rather than broadly aiming to enhance spiritual competence.

Collaboratively addressing the challenges healthcare professionals face through intervision meetings was found to be essential in supporting them in complex situations. Increased attention to the spiritual dimension enhances professionals’ awareness of ethical dilemmas and the need for reflection. These finding align with recent research on integrating attention for meaning in life and spirituality in home care [30]. We propose that fostering both personal and collective awareness of the spiritual dimension should be reinforced by institutional frameworks and work processes.

Qualitative observations revealed overlap between the subscales ‘Personal support and patient counselling’ and ‘Attitudes’. The findings indicate that ‘Personal support and patient counselling’ encompasses more than discussing residents’ spirituality, as is the primary focus of the questionnaire. Our observations point to the need for an alternative assessment tool that better captures the lived experiences in long-term care. A German study on spiritual care competences (*N* = 91) showed promising results using the Spiritual Care Competency Questionnaire (SCCQ) [27, 31]. The Dutch version of the SCCQ was validated among multi-professional mental healthcare-teams (*N* = 730), suggesting potential for broader application pending further research [32].

Importantly, the intervention demonstrated that pairing a spiritual caregiver with a vocational trained nurse or certified nurse assistant was highly effective. This collaboration helped balance theoretical and practical aspects of the spiritual dimension. Based on this finding, we suggest further development of train-the-trainer programs for paired professionals. Expanding the role of spiritual caregivers alongside other healthcare workers would strengthen efforts to integrate spirituality into daily practice, bridging the gap between theoretical knowledge and applied skills in daily practice.

## Strengths and limitations

To our knowledge, this is the first study to detail the specific skills, competencies and practices healthcare professionals employ to incorporate to the spiritual dimension of residents in a nursing home in daily practice. The study was conducted after a thorough process of co-creation with different stakeholders, strengthening organizational commitment. However, certain limitations must be acknowledged. First, the reflections are subjective in nature and the main researcher combined this study with performing the intervention, which might have caused bias. However, this risk was mitigated through collaboration with multiple coaches and data analysis by two independent researchers and regular consensus meetings. Secondly, the reliance on participants’ self-assessments presents potential bias in evaluating competency development. The SCCS measure was adapted for use in a long-term care setting. As a result, the validity and reliability of the adapted SCCS in this context may be limited. Additionally, the reduced number of post-test participants may have limited the strength of the comparative analysis and the generalizability of the quantitative findings. Furthermore, the interview quotes were translated into English, which might have caused linguistic bias. Another potential limitation of this study is the absence of a control group, which limits the ability to attribute observed effects solely to the intervention. This choice was deliberate and based on the nature of the research design. Given the multicomponent structure of the intervention—combining training sessions with individualized on-the-job coaching—it would have been methodologically and practically challenging to isolate components or apply a randomized controlled approach within just one nursing home setting. Nonetheless, future studies might consider a controlled or comparative design to further examine and validate the effects of similar interventions on spiritual care competencies. Such designs might include stepped-wedge trials, in which the intervention is rolled out sequentially across units, or matched comparison studies involving similar facilities without the intervention. These approaches would allow for more robust causal inference while respecting the ethical and logistical constraints of the care setting, providing additional insights into the effectiveness and generalizability of specific components of the intervention. Finally, the intervention was conducted in a single Dutch nursing home with a small sample size, and generalizability to other healthcare settings is unknown.

## Conclusions

Training, intervision meetings and coaching on the job significantly increased the self-reported spiritual care competence of the participating healthcare professionals in a nursing home. Individual on-the-job coaching helped participants address personal inquiries, develop awareness of their spiritual skills in daily practice and their own role in contact with residents. Observations revealed key competencies in aligning, connecting and deepening conversations, suggesting a need to re-evaluate the significance of ‘daily spirituality’ and the role of healthcare professionals in addressing the spiritual dimension. This study highlights how routine actions provide vital opportunities to engage with residents’ existential and spiritual concerns. Importantly, findings emphasize the necessity of considering the impact of the spiritual dimension in daily practice rather than solely striving for increased competence. Furthermore, training in pairs combining a spiritual caregiver with another healthcare professional proved effective in balancing theoretical and practical aspects of the spiritual dimension. We conclude that interventions incorporating both collective and individual learning foster the integration of the spiritual dimension in the daily practice of nursing home care.

## Data Availability

Sequence data that support the findings of this study have been deposited: 10.17026/LS/S6KTSN.

## Abbreviations

SCCS: Spiritual Care Competency Scale
SCCQ: Spiritual Care Competency Questionnaire

## Footnotes

1 https://www.cbs.nl/nl-nl/longread/statistische-trends/2023/arbeidsmarktprofiel-van-zorg-en-welzijn-in-2022/3-zorgmedewerkers.
